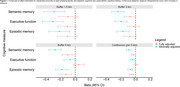# Residential proximity to a lead releasing facility is associated with cognition in KHANDLE and STAR cohorts

**DOI:** 10.1002/alz70860_103527

**Published:** 2025-12-23

**Authors:** Scarlet M Cockell, Kelly M. Bakulski, Ai‐Lin Tsai, Chinomnso Okorie, Stacey Alexeeff, Rachel A. Whitmer, Paola Gilsanz, Kathryn C Conlon

**Affiliations:** ^1^ University of Michigan, Ann Arbor, MI, USA; ^2^ Kaiser Permanente Northern California Division of Research, Pleasanton, CA, USA; ^3^ University of California, Davis, Davis, CA, USA

## Abstract

**Background:**

Environmental chemical exposures are potentially modifiable risk factors for dementia. While lead is a well‐documented early life neurotoxicant, pertinent time periods for exposures and their contribution to cognition in later life requires further investigation, particularly for diverse cohorts.

**Method:**

In a multi‐ethnic sample of participants from two harmonized cohorts (Kaiser Health Aging and Diverse Life Experiences (KHANDLE), Study of Healthy Aging in African Americans (STAR); *n* = 2,409), we assessed the relationships between residential proximity to a lead releasing facility, measured through the Toxics Release Inventory, with domain‐specific baseline cognition. Executive function, verbal episodic memory, and semantic memory were measured using the Spanish and English Neuropsychological Assessment Scales. We evaluated distance to the nearest lead releasing facility as a continuous measure as well as categorically (buffers with radii of 1.5 km, 3km, 6km) two years before cognitive testing. Linear regression models were adjusted for age at cognitive testing and cohort (minimally adjusted) and further adjusted for sex, race/ethnicity, income, education, and marital status (fully adjusted).

**Result:**

Average age at cognitive assessment was 74 years (SD=8), 62% were female, 48% identified as Black, 17% as Asian, 14% as LatinX, and 20% as Non‐Hispanic White. The average distance between residence and lead releasing facility was 6.6 km (SD=6.3). Every 5km decrease in residential distance from a lead releasing facility was associated with ‐0.07 lower verbal episodic memory (95% CI: ‐0.04, ‐0.10) and ‐0.09 lower semantic memory (95% CI: ‐0.06, ‐0.12) scores two years later. Living within a 6km buffer of a lead releasing facility was associated with ‐0.18 lower episodic memory (95% CI: ‐0.27, ‐0.10) and ‐0.27 lower semantic memory (95% CI: ‐0.36, ‐0.19) two years later. Point estimates were attenuated in fully adjusted models (Figure 1).

**Conclusion:**

Residential proximity to a lead releasing facility may be associated with poorer cognition among older adults in a diverse cohort, and comprehensive understanding of environmental factors related to dementia is a critical step to advance disease prevention.